# Investigation of Silver- and Plant Extract-Infused Polymer Systems: Antioxidant Properties and Kinetic Release

**DOI:** 10.3390/ijms252312816

**Published:** 2024-11-28

**Authors:** Magdalena Bańkosz, Bożena Tyliszczak

**Affiliations:** 1 Department of Material Engineering, Faculty of Materials Engineering and Physics, CUT Doctoral School, Cracow University of Technology, 31-864 Kraków, Poland; 2 Department of Material Engineering, Faculty of Materials Engineering and Physics, Cracow University of Technology, 31-864 Kraków, Poland

**Keywords:** compound release kinetics, antioxidants, porosity, physical and chemical properties, material characterization, vapor permeability, roughness

## Abstract

This study evaluated the impact of silver particles, suspended in *Arnica montana* flower extract, on the physicochemical characteristics and release dynamics of antioxidant compounds in PVP (polyvinylpyrrolidone)-based hydrogel systems. The hydrogels were synthesized via photopolymerization with fixed amounts of crosslinker (PEGDA) and photoinitiator, while the concentration of the silver-infused extract was systematically varied. Key properties, including the density, porosity, surface roughness, swelling capacity, and water vapor transmission rate (WVTR), were quantitatively analyzed. The results demonstrated that increasing the silver content reduced the hydrogel density from 0.6669 g/cm^3^ to 0.2963 g/cm^3^ and increased the porosity from 4% to 11.04%. The surface roughness parameters (Ra) rose from 8.42 µm to 16.33 µm, while the WVTR increased significantly from 65.169 g/m^2^·h to 93.772 g/m^2^·h. These structural changes directly influenced the release kinetics of antioxidant compounds, with kinetic modeling revealing silver-dependent variations in the evaluated release mechanisms. This innovative approach of integrating silver particles and plant-derived antioxidants into hydrogels highlights a novel pathway for tailoring material properties. The observed enhanced porosity and moisture regulation underscore the hydrogels’ potential for biomedical applications, particularly in wound care, where controlled moisture and antioxidant delivery are critical. These findings provide new insights into how silver particles modulate hydrogel structures and functionalities.

## 1. Introduction

Silver particles are widely utilized in various applications for their antimicrobial and antioxidant properties. Motelica et al. have highlighted their innovative use in biodegradable and antimicrobial packaging systems, such as alginate-based films enhanced with silver nanoparticles (AgNPs) and essential oils. These films demonstrated strong antibacterial activity and potential for preserving perishable goods, such as cheese, by extending their shelf life and reducing microbial contamination [1,2,3]. The specific size and shape of these particles are critical, as they strongly influence their behavior and effectiveness. The synthesis approach and reaction conditions, such as the temperature, reactant concentration, pH, and synthesis duration, dictate the final particle dimensions. Studies show a wide size range of sizes, with Sadeghi et al. [4] obtaining particles between 10 and 50 nm while Murugan et al. [5] achieved larger, micrometer-sized particles using plant-based extracts.

Biosynthesis methods using plant extracts are emerging as eco-friendly, low-cost alternatives to traditional chemical synthesis. These methods bypass the need for toxic chemicals and leverage plant-derived compounds—such as phenols, flavonoids, organic acids, and terpenes—that naturally reduce and stabilize silver particles [6,7,8]. For instance, Ahmed et al. [9] synthesized silver particles ranging from 100 nm to several micrometers using Azadirachta indica extract, with the particle size being influenced by the extract concentration and reaction duration. This approach underscores the potential of plant-based synthesis for producing silver particles with tailored properties.

Research into biosynthesizing silver nanoparticles through plant extracts has shown their broad utility across various biological fields. For example, Dwivedi et al. [10] synthesized AgNPs using *Chenopodium album* leaf extract. On the other hand, Deenadayalan et al. successfully created AgNPs using *Chenopodium album* leaf extract, while Deenadayalan et al. [11] employed cymbopogon citratus (lemongrass) extract to generate nanoparticles with notable antioxidant properties. These findings support the potential of the plant-based synthesis of silver nanoparticles for applications in areas such as healthcare, food processing, and cosmetics.

Among the promising plant sources for this biosynthesis approach is *Arnica montana*, recognized for its therapeutic qualities. Extracts from *Arnica montana* flowers are rich in bioactive substances, including phenols, flavonoids, and other compounds with strong anti-inflammatory, antibacterial, and antioxidant effects, making them an excellent choice for producing silver nanoparticles [12,13,14]. Zitek et al. [15] explored the antioxidant capabilities of *Arnica montana* flower extracts, demonstrating their effectiveness in neutralizing free radicals and stabilizing silver particles. This research also emphasized that factors like the extract concentration and reaction time greatly affect the properties and dimensions of the synthesized nanoparticles.

The investigation of plant extracts for nanoparticle synthesis has gained particular importance due to their intrinsic antioxidant properties. Antioxidants play a crucial role in scavenging reactive oxygen species (ROS), which are implicated in various pathological conditions, including inflammation, cancer, and neurodegenerative disorders [16,17,18]. In the context of nanoparticle synthesis, antioxidants not only aid in reducing the number of metal ions but also provide an additional layer of bioactivity to the resulting materials. Different studies have demonstrated that nanoparticles synthesized using plant extracts exhibit enhanced antioxidant activities, contributing to their efficacy in wound healing and anti-cancer therapies [19,20]. Next, work by Mittal et al. highlights how bio-inspired silver nanoparticles synthesized using plant extracts exhibited superior antimicrobial and antioxidant properties compared to their chemically synthesized counterparts, making them highly suitable for biomedical applications [21]. Moreover, the antioxidant-rich matrices of plant-derived nanoparticles play a significant role in stabilizing cellular environments during oxidative stress. These findings underline the dual functionality of plant extracts as both bioreductants and bioactive agents, further enhancing their value in the development of advanced biomaterials [22]. The application of such antioxidant-enriched materials is especially relevant in wound care, where oxidative stress significantly hinders the healing process. By incorporating bioactive nanoparticles into wound dressings, it is possible to neutralize ROS, reduce inflammation, and prevent microbial infections, thereby creating an optimal environment for tissue regeneration [23,24]. Additionally, the release kinetics of these bioactive compounds is a critical factor that warrants further study, as their controlled and sustained release would ensure prolonged therapeutic efficacy. Advances in this area could lead to next-generation biomaterials that integrate antioxidative and antimicrobial properties with precision drug delivery capabilities, offering tailored solutions for chronic and acute wound management [25,26].

Designing materials that are capable of releasing antioxidant-active substances in a controlled and sustained manner is of paramount importance in biomedical applications. Among these, biohydrogels have emerged as highly promising systems for managing bioactive compounds due to their unique physicochemical properties and biocompatibility. Biohydrogels are hydrophilic polymer networks that can retain significant amounts of water, mimicking the extracellular matrix, which makes them ideal for delivering therapeutic agents while maintaining a moist environment that is conducive to healing [27,28,29]. Their tunable porosity and degradation rates enable precise control over the release kinetics of encapsulated antioxidants, ensuring their prolonged bioactivity and reducing the need for frequent reapplication [30]. Furthermore, their ability to incorporate plant-derived nanoparticles or extracts rich in antioxidants provides an additional layer of functionality, combining antioxidative, antimicrobial, and wound-healing properties in a single platform [31,32].

This investigation aims to characterize the antioxidant capabilities of polymeric matrices embedded with silver particles synthesized from *Arnica montana* flower extracts. The base material was a hydrogel based on polyvinylpyrrolidone (PVP), a synthetic polymer that is widely recognized for its excellent biocompatibility, non-toxicity, and favorable physicochemical properties. PVP is particularly attractive for biomedical applications due to its high water-retention capacity, which promotes cell proliferation and maintains a moist wound environment that promotes healing [33,34]. Its chemical stability and ease of functionalization allow for the incorporation of various bioactive compounds, including antioxidants, into it, making it an ideal candidate for advanced hydrogel systems [35]. Diverging from the focus on nanoparticles of many previous studies, this research employed synthesis conditions that yielded micrometer-sized silver particles [36,37,38,39]. This study emphasizes the kinetic modeling of antioxidant compound release by utilizing the Folin–Ciocalteu reagent reduction assay, a standardized method for quantifying antioxidant activity. Additionally, comprehensive characterization of the physicochemical properties of the studied materials, including their density and porosity, was conducted. This study further examines the influence of compositional variations on barrier properties such as the vapor permeability and swelling capacity, facilitating a deeper understanding of moisture interactions within the materials. Surface roughness profiling was also performed to elucidate microstructural attributes. This extensive analysis is critical for evaluating the applicability of these systems, particularly in biomedical contexts and as protective materials against oxidative stress.

## 2. Results and Discussion

### 2.1. Characteristics of Silver Particles

**Figure 1:**
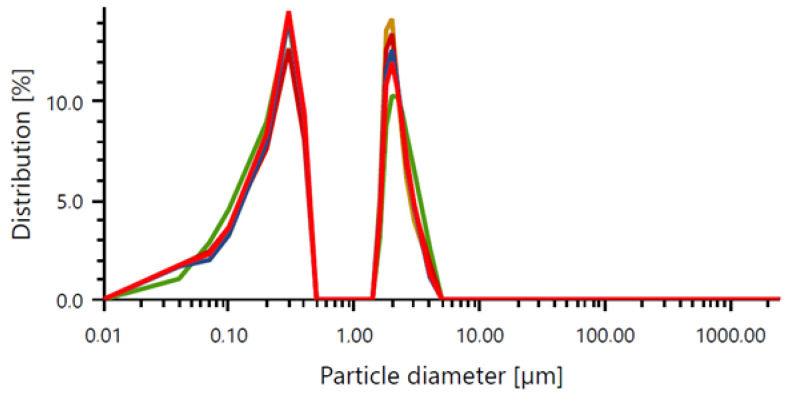
Particle size distribution made using five repetitions (each color line corresponds to one repetition).

The first step in the analysis was to characterize the suspension of silver particles. For this purpose, a particle size analysis was performed, the results of which are presented in Table 1 and Figure 1.

The analysis of silver particles synthesized with *Arnica montana* extract demonstrated a notable range of particle sizes, with an average particle size of 0.7816 µm, confirming that the particles produced were predominantly in the micrometer range. Further examination of the particle size distribution provided detailed insights: the D_10_ value was 0.04651 µm, indicating that 10% of the particles measured as being below this threshold; the D_50_, or median value, was 0.2351 µm, meaning that half of the particles were smaller than this size; and the D_90_ value was 2.211 µm, indicating that 90% of the particles fell below this size, though a small fraction of larger particles was observed. The standard deviations for these measurements were recorded as 0.00741 µm (D_10_), 0.00310 µm (D_50_), 0.07869 µm (D_90_), and 0.02798 µm for the mean particle size. The relatively low standard deviations for the D_10_ and D_50_ reflect a stable distribution among the smaller particles, whereas the higher deviation in D_90_ suggests greater size variability in the larger particles, potentially due to aggregation during synthesis.

Overall, the results indicate a wide particle size distribution, with a dominance of particles in the micrometric size range. Such variation may have implications for the functional properties of the materials, such as its antioxidant activity or bioactivity, as well as the potential applications of these materials in various fields, including biomedicine and materials technology. It is important to emphasize that the size of the particles, particularly in the micrometer range, significantly influences their functional properties. Particles of this size exhibit greater physical and chemical stability compared to nanoparticles, reducing the risk of agglomeration and enhancing their durability across various environments. Additionally, their large specific surface areas facilitate effective interactions with other substances, which is crucial in applications such as adsorption, catalysis, and drug delivery systems. In the biomedical field, micrometer-sized particles can offer a more predictable release profile of active substances, making them promising candidates for targeted therapies. In materials technology, their size can contribute to improved mechanical, thermal, or optical properties, enabling their use in advanced composites and protective coatings. For instance, mesoporous silica nanoparticles (MSNs) are widely used in drug delivery due to their high surface area and tunable pore sizes, which allow for efficient drug loading and controlled release [40]. Moreover, the stability of nanoparticle dispersions is influenced by the particle size, with larger particles exhibiting reduced tendencies to agglomerate, thereby maintaining their functional properties over longer time periods [41]. In materials science, the sizes of nanoparticles affect their reactivity and stability, with larger particles often displaying an enhanced stability and specific surface area, which are beneficial for various applications [42].

Obtaining particles in the micrometer size range may result from conducting the reaction at room temperature. The findings of other researchers confirm the relationship between the reaction temperature, the particle size, and the mechanism of their formation. For example, in the study by Liu et al., the focus was on the influence of temperature on the nucleation kinetics (kinetic constant k_1_) and particle growth (kinetic constant k_2_) in the chemical synthesis of metal nanoparticles, particularly silver nanoparticles (AgNPs). Based on a microscopic quantitative kinetics analysis, it was found that higher temperatures promote both nucleation and particle growth, as both the k_1_ and k_2_ increase with a rising temperature. However, differences in their behavior were observed—the k_1_ increases sharply above 80 °C, while the k_2_ rises almost linearly with the temperature. When sufficient Ag^+^ ions are present, the particle size increases with the growth of k_2_, while the k_1_ mainly affects the number of nanoparticles formed. In contrast, under insufficient Ag^+^ precursor conditions, rapid nucleation at high temperatures depletes the available silver ions, limiting particle growth and reducing the particle size. The increase in particle size between 70 and 80 °C, followed by a sharp decrease above 80 °C, is due to the rapid rise in the k_1_ rather than a decrease in the k_2_ [43].

The synthesis of micrometer-scale silver particles at room temperature presents notable advantages. Conducting the reaction at lower temperatures enables milder, eco-friendly conditions, which decreases energy usage and minimizes the likelihood of uncontrolled nucleation. This approach supports sustainable development goals by reducing the carbon emissions resulting from the process and improving the resulting material stability. Moreover, micrometer-sized particles can provide benefits such as a more gradual release of bioactive compounds and enhanced structural integrity.

**Figure 2:**
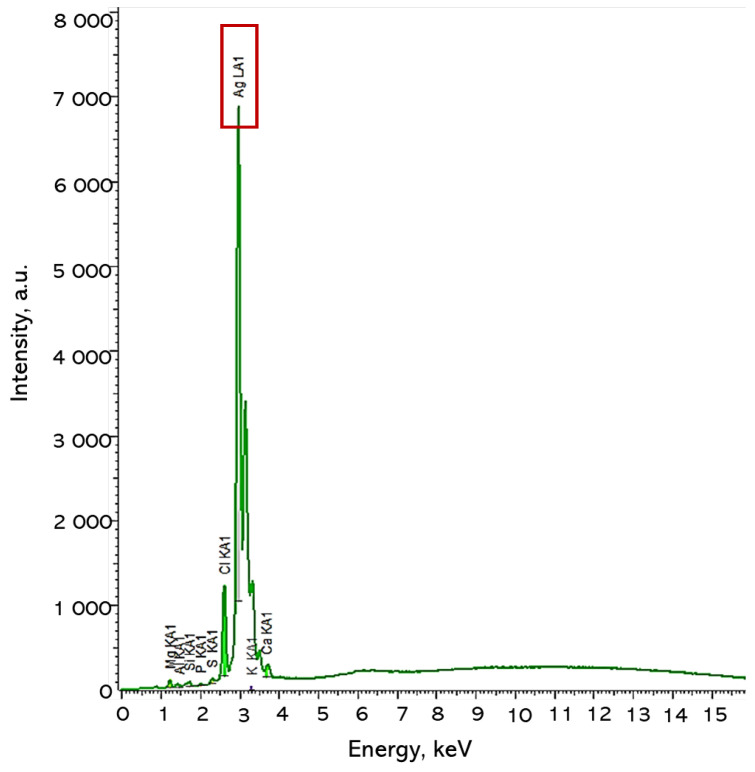
XRF results of Ag suspension.

XRF analysis was then carried out determining the elemental composition of the suspended silver particles in the plant extract. The results are presented in Table 2 and Figure 2.

XRF analysis revealed that the silver particles suspended in a fresh portion of plant extract are composed predominantly of silver (Ag), with a concentration of 96.03%. This high level of silver confirms the synthesis process’s efficiency in generating silver particles. Minor quantities of other elements—such as calcium (Ca), chlorine (Cl), silicon (Si), aluminum (Al), magnesium (Mg), sulfur (S), and phosphorus (P)—were detected, with calcium showing the highest concentration at 1.57%. These trace elements are attributed to the natural constituents of the plant extract in which the particles remain suspended. Although detectable, these elements are present at such low levels that they are unlikely to influence the primary properties of the silver particles, which are predominantly governed by the silver content, with other elements serving as minimal impurities from the extract.

### 2.2. FT-IR Infrared Spectroscopy Analysis

**Figure 3:**
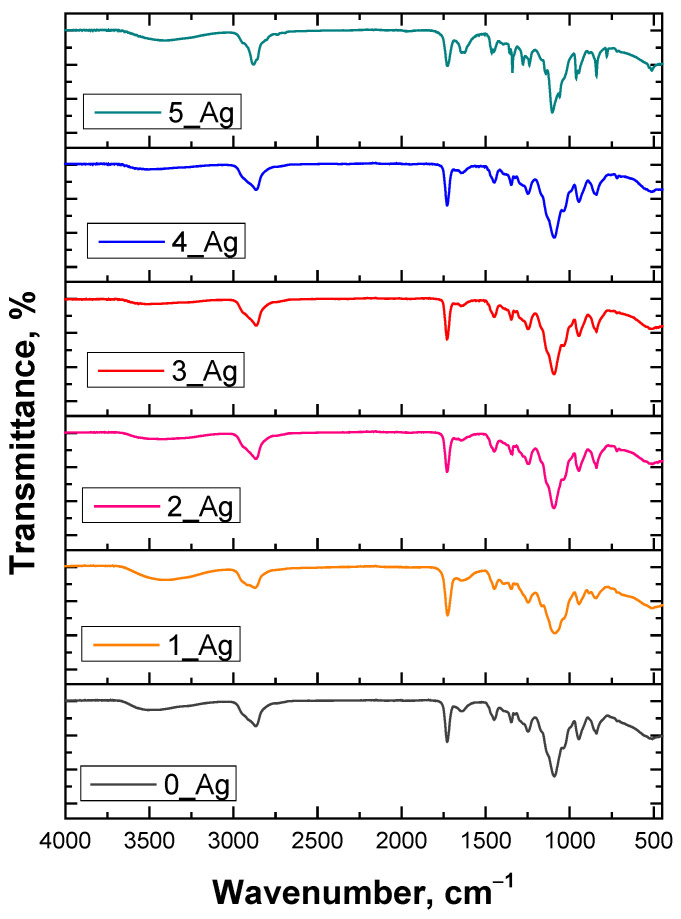
FT-IR spectra of hydrogel materials.

Infrared spectroscopy analysis was carried out to evaluate possible changes in the spectra of the particle suspension-modified materials relative to the base material. The results of the analysis are presented in Figure 3.

FT-IR analysis of the hydrogel samples containing varying concentrations of silver particles and plant extract revealed distinct absorption bands corresponding to the primary components of the material: the PVP polymer, the PEGDA crosslinker, the photoinitiator (2-hydroxy-2-methylpropiophenone), and the plant extract with the silver suspension. 

A detailed summary of these characteristic absorption bands is provided in Table 3.

In this analysis, the bands in the 3400–3500 cm^−1^ range may come not only from hydroxyl groups from the water and PVP, but also from phenols present in the plant extract, which are known to have O–H groups. In addition, the bands in the range of 1650–1700 cm^−1^, typical of carbonyl bond stretching (C=O), can be associated with both the PVP and PEGDA, as well as with flavonoids and other organic compounds from the extract. Also, in the 1000–1100 cm^−1^ range, bands associated with alcohol and phenolic groups from the extract, which can affect the structural properties of the hydrogel, can be observed. An increase in the amounts of plant extract and silver particles does not cause significant changes in the position of the bands, suggesting that these additives do not significantly affect the chemical structures of the main hydrogel components.

### 2.3. Analysis of Physicochemical Properties

**Figure 4:**
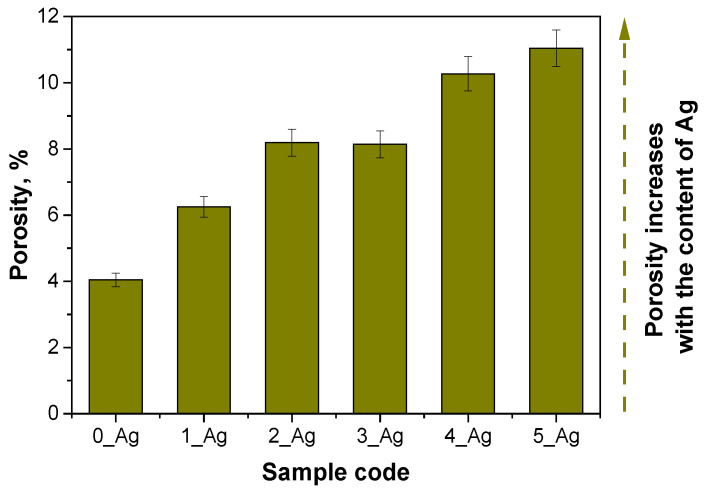
Porosity of polymeric systems without and with Ag suspension (the standard deviation is marked on the graph, the number of repetitions = 3.

**Figure 5:**
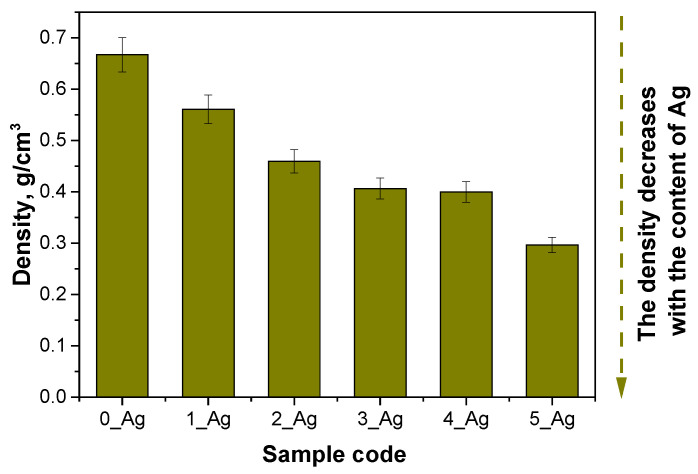
Density of polymeric systems without and with Ag suspension (the standard deviation is marked on the graph, the number of repetitions = 3.

**Figure 6:**
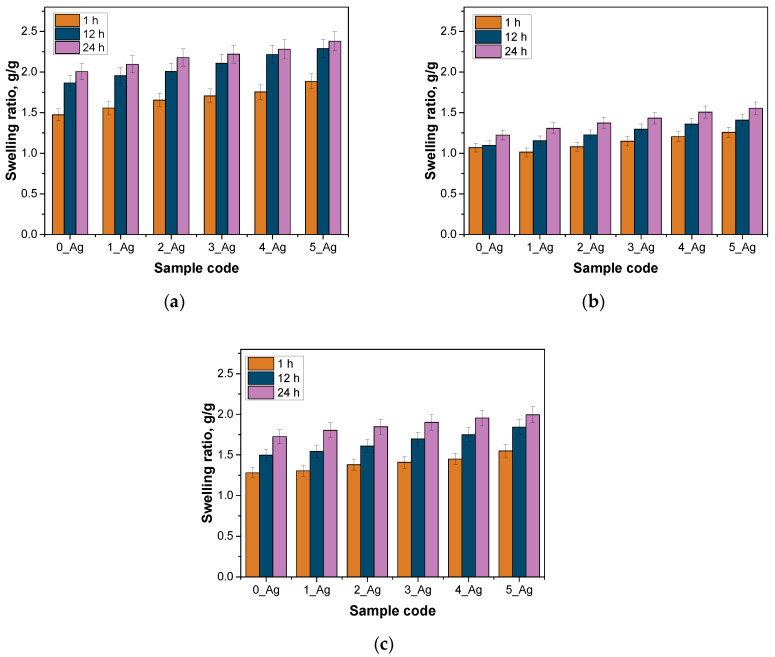
Sorption capacity of polymer systems in distilled water (**a**), SBF (simulated body fluid) (**b**), and Ringer’s fluid (**c**).

A detailed physicochemical analysis of these hydrogels was then carried out, including measurements of their density, porosity, and swelling capacity. These properties are key to understanding the material’s internal structure, stability, and interaction with water, which have implications for potential applications such as the controlled release of active ingredients or biomedical applications, where the porosity and swelling are important for transporting substances through the hydrogel matrix. The results of these measurements are presented in Figure 4, Figure 5 and Figure 6. The results of the statistical analysis of sorption capacity are presented in Table 4.

The hydrogel analysis findings indicate that the photopolymerization process, involving fixed concentrations of crosslinker and photoinitiator along with progressively increasing amounts of aqueous extract containing silver particles, significantly influenced the material structure of the hydrogels, including parameters such as the density, porosity, and sorption capacity. The reference sample (0_Ag), which lacked silver-containing extract, exhibited the highest density, reflecting a highly compact polymer network (d = 0.6669 g/cm^3^). This tightly crosslinked network minimized the available space for liquid retention, resulting in the lowest levels of porosity and liquid absorption capacity.

In samples with progressively larger volumes of silver-containing extract, the crosslinker concentration remained constant. This increase in aqueous suspension volume likely led to a partial dilution of the reaction mixture, which may have decreased the crosslinking efficiency. Consequently, the polymer networks in samples with higher silver contents (e.g., 4_Ag and 5_Ag) were less densely packed, leading to reduced sample densities (d = 0.2963 for sample 5_Ag). This looser network structure created additional spaces between polymer chains, resulting in greater porosity. The enhanced porosity allowed increased liquid penetration into the hydrogel’s interior, which in turn raised its sorption capacity (swelling) and improved its water vapor permeability.

The base sample (0_Ag), which lacked the silver-containing extract, exhibited the highest density, 0.6669 g/cm^3^, indicating a tightly packed polymer network that limited the space that was available for liquid uptake. This compact structure was reflected in the sample’s low porosity (around 4%) and restricted sorption capacity, allowing it to absorb only a minimal amount of water. With the progressive addition of the extract from samples 1_Ag to 5_Ag, the density systematically decreased, reaching 0.2963 g/cm^3^ in sample 5_Ag. This reduction in density suggests a decrease in the crosslinking intensity, leading to an increase in porosity, which reached 11.04% in sample 5_Ag. The increased porosity enabled easier water entry, resulting in a marked rise in the sorption capacity (α = 1.4732 for sample 0_Ag; α = 1.8853 for sample 5_Ag in distilled water after 1 h). The statistical analysis of the sorption ability after 1 h demonstrated that all investigated factors and their interactions have significant effects. The type of incubation fluid exhibits a highly significant impact on the sorption ability, as indicated by an exceptionally low *p*-value (*p* = 2.45 × 10^−28^), suggesting that the fluid type strongly influences the swelling behavior of hydrogels. Similarly, the composition of the sample also significantly affects its sorption ability (*p* = 4.44 × 10^−16^), indicating that changes in the sample structure or formulation lead to measurable differences in its performance. Furthermore, the interaction between the type of incubation fluid and the sample composition is significant (*p* = 0.01049), demonstrating that the effect of one factor depends on the level of the other. These findings underscore the complex relationship between a hydrogel’s composition and the environmental conditions, as well as their combined influence on the hydrogel’s functional properties.

The observed relationship between the density, porosity, and sorption aligns logically: as the density decreases, the hydrogel network opens up, enhancing the liquid penetration, absorption, and water vapor permeability. Samples 4_Ag and 5_Ag, with the highest extract concentrations, demonstrated the greatest porosity and water absorption, supporting the hypothesis that adding an aqueous suspension contributes to loosening the polymer network structure. An inverse relationship between density and porosity has also been reported by Chen et al. Chavda et al. similarly highlighted correlations between crosslinking levels and the physical properties of porosity, density, and sorption capacity. Consistent with these findings, the arrangement of polymer chains within the matrix underlies a trend where a lower density corresponds to increased porosity and sorption properties [44]. Moreover, the results obtained in this study can be compared with the work of Tak et al., who analyzed the mechanical and functional properties of hydrogels containing *Bergenia stracheyi* extract. Although their research did not involve direct measurements of porosity, it was observed that the presence of the plant extract enhanced the swelling capacity of and imparted self-healing and adhesive properties to the hydrogels. The moderate swelling capacity indicated limited changes in the hydrogel structure, which contrasts with the findings of this study, where a progressive increase in silver content resulted in a significant rise in the porosity and sorption capacity [45]. In the work of Mittal et al. (2020), the focus was on developing hydrogels containing *Didymocarpus pedicellatus* extract and their ability to accelerate wound healing. The authors demonstrated that the studied hydrogels exhibited a high swelling capacity, reaching up to 1600%. It was also emphasized that the impregnation of the hydrogels with plant extract improved water distribution within the hydrogels’ matrices and enhanced their adhesive properties. Similarly to the present study, it was observed that changes in hydrogel structures, such as increased water absorption, are closely related to the modification of their polymer network through the introduction of active components [46].

### 2.4. Water Vapor Transmission Rate

**Figure 7:**
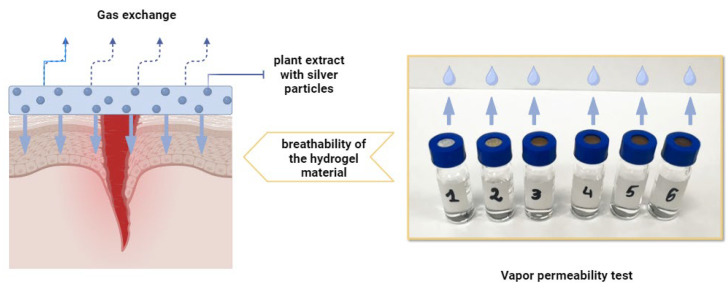
Scheme of water vapor permeability test.

**Figure 8:**
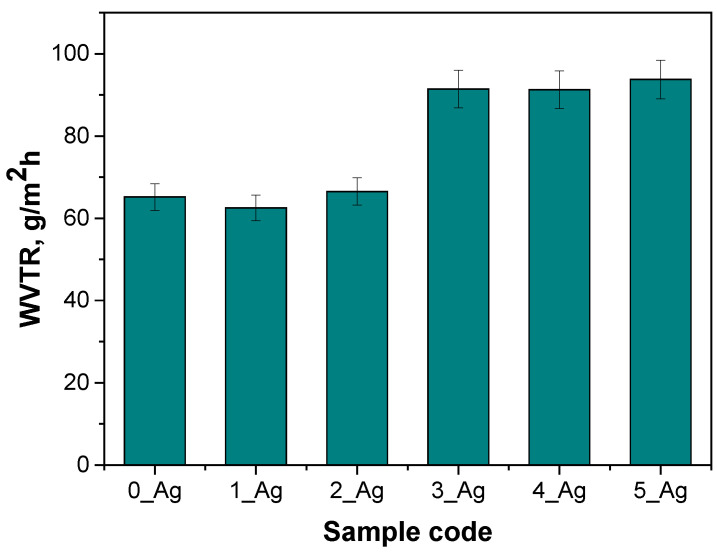
Results of water vapor permeability test (the standard deviation is marked on the graph, the number of repetitions = 3).

The purpose of the water vapor transmission rate (WVTR) analysis was to determine how the addition of silver particles in varying concentrations affects the barrier properties of hydrogels. The WVTR is a key parameter for materials used as wound dressings, as membranes, or in other applications where moisture regulation is crucial. An optimal WVTR ensures proper hydration and supports the healing process while protecting against excessive water loss and pathogen infiltration. The study scheme for the determination of the WVTR is presented in Figure 7, while the results are shown in Figure 8.

Figure 7 illustrates the scheme of the water vapor permeability test. On the left, a schematic representation of gas exchange in a dressing placed over a wound is presented, highlighting the role of the dressing in regulating moisture and facilitating evaporation. On the right, images of the samples used during the water vapor permeability test are shown, demonstrating the experimental setup and methodology for assessing this critical property.

The analysis of the results shows that the water vapor transmission rate (WVTR) significantly increased with the increasing content of silver particles in the hydrogels. The sample without silver (0_Ag) had the lowest WVTR, at 65.169 g/m^2^h, while the sample with the highest amount of silver (5_Ag) reached the highest WVTR, at 93.772 g/m^2^h.

For samples with lower silver contents (1_Ag and 2_Ag), the WVTR remained close to that of the control sample (without silver), suggesting that small amounts of silver particles do not significantly affect the water vapor permeability. Only at higher silver concentrations (samples 3_Ag, 4_Ag, and 5_Ag) was a noticeable increase in the WVTR observed, likely due to changes in the microstructure of the hydrogel. The addition of more silver may have affected the material’s porosity, increasing the number of pathways for water vapor transmission.

The density analysis additionally demonstrated that, with increasing silver particle contents, the hydrogel density declined, while the porosity rose. This reduction in density, along with the increase in porosity, aligns with our findings on water vapor transmission, indicating that greater porosity allows for easier moisture penetration through the material. These consistent results suggest that the addition of silver particles impacts the hydrogel’s internal structure, enhancing its porosity, which in turn leads to increased water vapor transmission. The observed decrease in density, paired with the heightened porosity and WVTR, provides a thorough understanding of how the silver particles influence the hydrogel’s physicochemical properties. Such changes in microstructure can be essential for optimizing hydrogels in applications such as wound dressings, where regulated moisture control and a suitable permeability are necessary. In parallel, Bahadoran et al. found that incorporating substances like alginate into PVA/sodium alginate hydrogels increased their porosity, leading to an enhanced water absorption capacity and elevated water vapor permeability. Likewise, our study’s findings of increased porosity and vapor permeability in samples with higher silver contents suggest similar structural adjustments, directly impacting the moisture permeability properties of the hydrogel [47].

High vapor permeability is particularly beneficial in fields such as biomedical engineering, especially in the development of wound dressings. Such dressings require controlled moisture regulation to promote wound healing by preventing excessive drying or maceration. Moreover, hydrogels with high water vapor transmission rates can be applied in drug delivery systems, where the material’s permeability ensures the controlled release of therapeutic agents. In food packaging, similar properties could be advantageous for maintaining the quality of perishable goods by allowing the release of excess moisture while maintaining an adequate barrier to microbial contamination. In wearable technology, hydrogels with optimized vapor permeability can be used as breathable, moisture-wicking components in sensors or smart textiles. The ability to fine-tune these properties by adjusting the silver content of hydrogels offers significant potential for creating materials tailored to diverse applications where moisture control is a critical factor. Pan et al. measured the moisture vapor transmission rates (MVTRs) of hydrogels composed of poly(vinyl alcohol), human-like collagen, and carboxymethyl chitosan. Their study highlighted that hydrogels with optimized pore structures facilitated adequate gaseous exchange, contributing to effective wound healing by preventing exudate accumulation and creating a suitable environment for tissue repair [48]. Next, Yang et al. similarly assessed the water vapor permeability of cellulose nanofibril-reinforced hydrogels, noting that their porous network allowed for controlled vapor transmission. This permeability ensured a balance between moisture retention and evaporation, which is crucial for promoting skin regeneration and preventing bacterial colonization. These findings underscore the role of water vapor permeability in enhancing the functionality of hydrogel-based wound dressings [49].

### 2.5. Evaluation of Surface Roughness and Microscopic Observations

**Figure 9:**
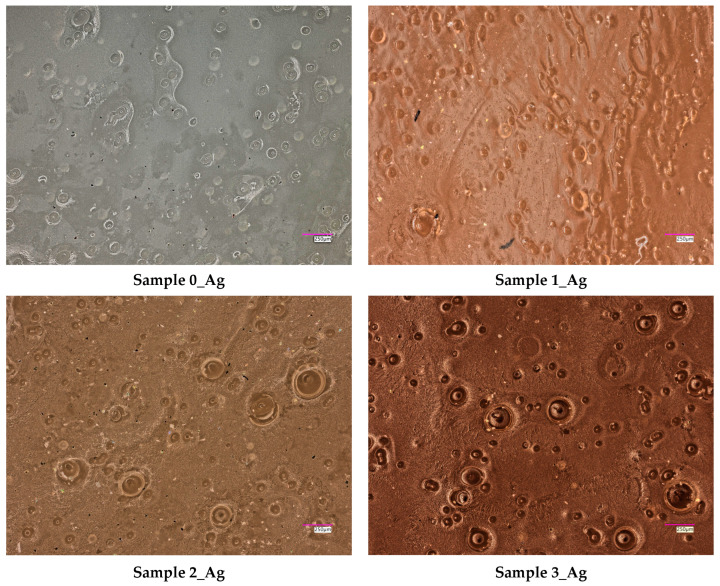

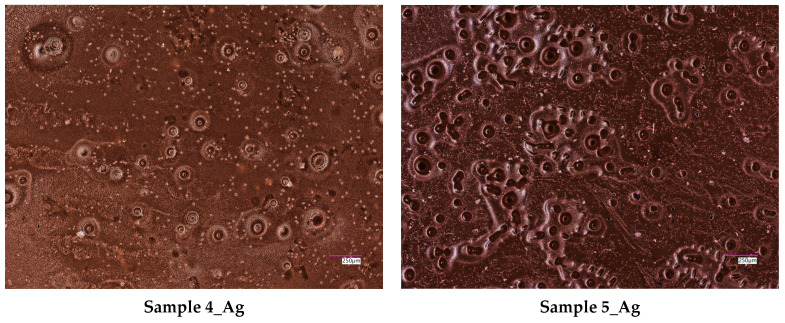
Results of microscopic observations of the surfaces of the obtained hydrogel samples.

Then, observations were carried out using a digital microscope. The obtained images are presented in Figure 9.

Based on the images presented in Figure 9, it can be observed that the surfaces of the polymer materials differ depending on the amount of silver nanoparticle suspension used during their synthesis. In samples where larger amounts of suspension were used, an increased number of pores is visible on the surface. For the control sample (without the addition of the suspension), pores were also observed, although to a much lesser extent. This phenomenon may be attributed to the use of a homogenizer during the synthesis of the samples, which influences the uniformity of the mixture while also introducing small air bubbles. In samples with larger amounts of suspension, the homogenization time was longer, which may have contributed to the formation of a greater number of pores or the entrapment of more air bubbles in the matrix. The presence of pores on the material’s surface is likely associated with the mixing process, and their quantity appears to be proportional to the volume of suspension used in the synthesis. This could have a significant impact on the functional properties of the materials, such as their porosity, permeability, or mechanical properties.

**Figure 10:**
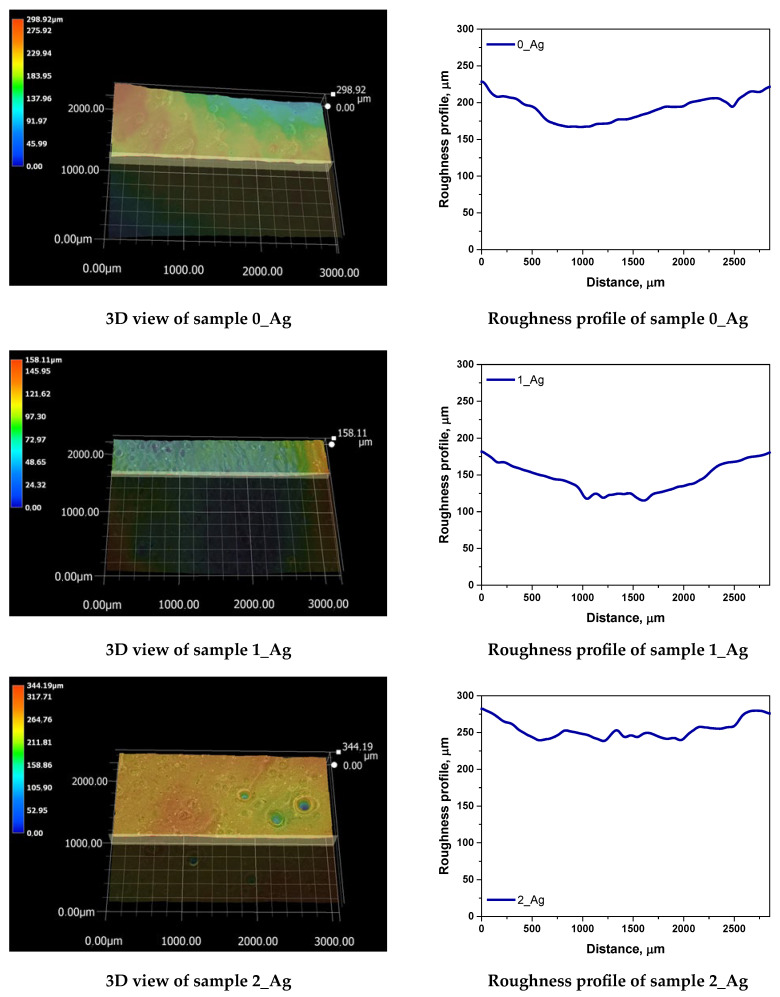

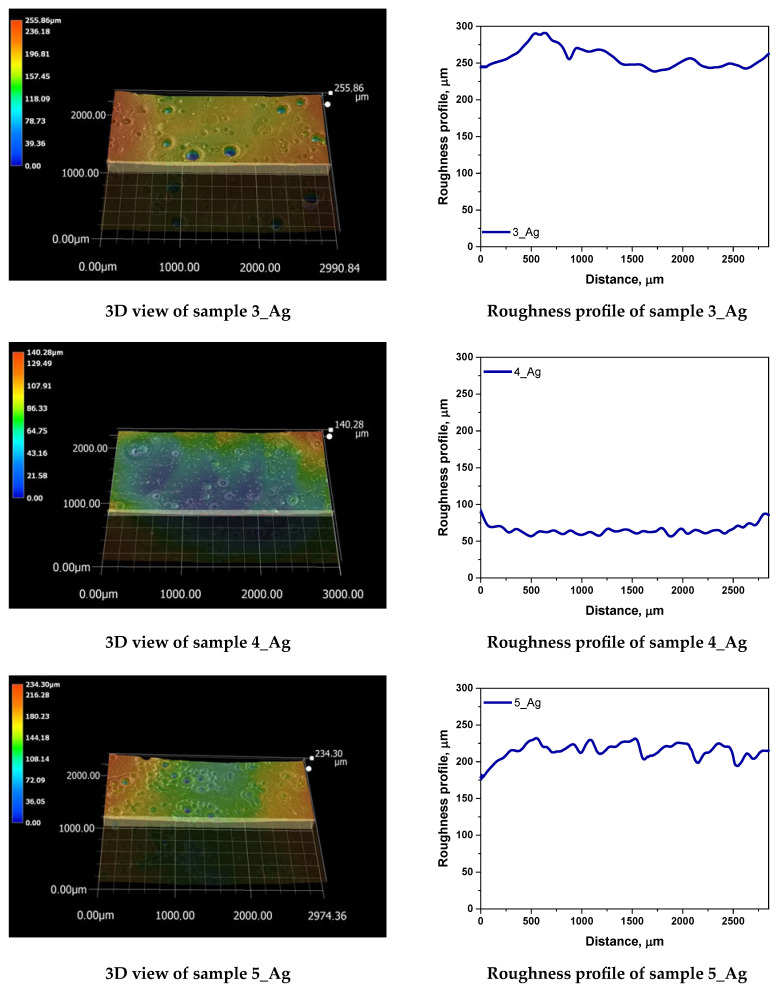
3D view and roughness profiles of the obtained polymer systems.

We conducted a roughness profile analysis, aiming to evaluate the impact of increasing concentrations of silver particles suspended in the plant extract on the resulting hydrogel surface’s roughness. Parameters such as the arithmetic mean roughness (Ra) and maximum roughness (Rz) offer insights into the surface texture, which is crucial for understanding a material’s functional properties, including its water interaction, adsorption capacity, and potential biological interactions. The analysis results are illustrated in Figure 10 and Table 5 below.

The analysis results indicate that, as the concentration of silver particles in the samples increases, the roughness parameters exhibit a variable trend. In the base sample 0_Ag (without silver), the Ra and Rz values are the lowest, measured at 8.42 and 46.47, respectively. With the progressive addition of silver particles in samples 1_Ag through 5_Ag, both the Ra and Rz values increase, reaching their peaks in sample 5_Ag, where the Ra is 16.33 and the Rz is 59.78. The highest roughness, indicated by Ra = 16.33, is observed in the sample with the greatest silver content (5_Ag), suggesting that increasing silver and plant extract amounts contribute to a more irregular hydrogel surface texture. This roughness increase may be attributed to the aggregation of silver particles on the hydrogel’s surface, resulting in more pronounced topographical features. Furthermore, the variation in Rz values implies that the maximum surface height differences are greater in samples with a higher silver content, although the increase is not strictly uniform.

For example, data from the literature also suggest that higher concentrations of silver nanoparticles within a hydrogel matrix contribute to increased surface roughness, likely due to the formation of particle aggregates on the hydrogel surface, similar to what was observed in samples 3_Ag and 5_Ag [50]. Likewise, a study by Moradian et al. on silver nanoparticle composites found that adding silver raised the material roughness. Specifically, nanoparticle aggregation on the surface contributed to greater topographical complexity, impacting both the material’s mechanical and functional properties [51]. Enhanced roughness can improve a material’s biophysical characteristics, such as its wettability and cellular interactions, which are essential for biomedical hydrogel applications, including as wound dressings and drug delivery systems.

### 2.6. Incubation in Simulated Body Fluids

**Figure 11:**
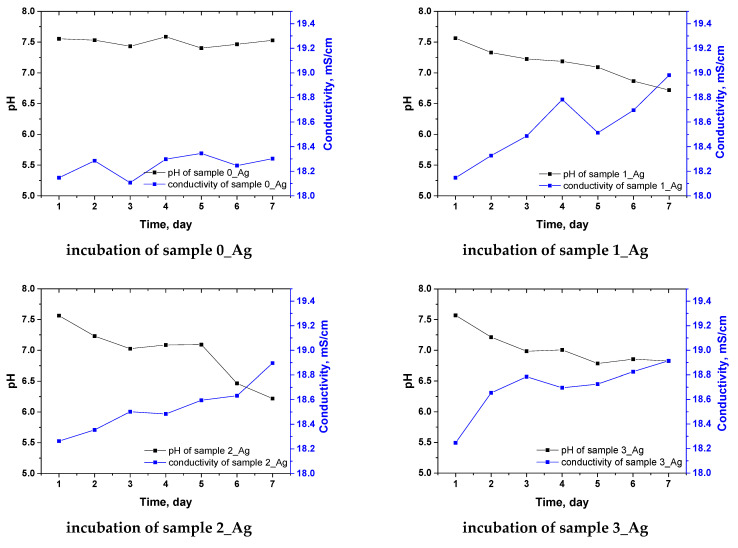

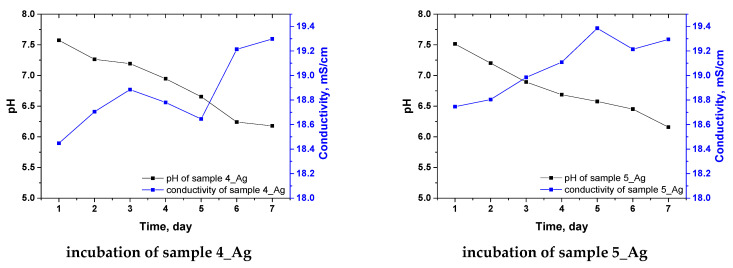
Incubation of hydrogel samples: pH changes (black line), conductivity changes (blue line).

A 7-day incubation study of hydrogels in simulated body fluid (SBF) offered valuable insights into pH fluctuations and ionic conductivity changes in the presence of hydrogels containing varying amounts of *Arnica montana* extract with suspended silver particles. These findings highlight notable alterations in the properties of the incubation fluid that are directly linked to the release of compounds from the plant extract and their interactions with the surrounding environment. The incubation outcomes are depicted in Figure 11.

In the control samples (0_Ag), which lacked both extract and silver particles, the pH remained stable at approximately 7.4–7.5 throughout the incubation period. This stability indicates that hydrogels without added modifiers (extract with silver particles) do not impact the ionic balance of SBF solution, suggesting low reactivity and the absence of acidic or basic compounds’ release. In contrast, samples containing the extract with silver particles showed a gradual decrease in pH, which intensified with higher extract concentrations. The most substantial pH drop was observed in sample 5_Ag, where the pH fell to 6.157 after 7 days, a marked decrease compared to the control. This pH reduction likely results from the release of bioactive compounds within the extract, particularly acidic groups such as phenolic acids, which are capable of releasing H^+^ ions. *Arnica montana* extract is rich in such acidic compounds, contributing to the medium’s acidification as they leach from the hydrogel matrix. The higher the extract concentration (from 1_Ag to 5_Ag), the greater the presence of these acidic compounds, leading to a progressively more pronounced pH decrease.

Alongside the pH variations, an increase in ionic conductivity was noted in samples containing the silver particle extract, corresponding to the rising release of ions from the hydrogels. In the control sample (0_Ag), the conductivity remained nearly constant, reflecting its stability and lack of reactivity with the SBF solution, similar to the stable pH which was also observed. In samples with added extract and silver particles, the conductivity progressively rose with both the incubation time and extract concentration. For sample 5_Ag, the conductivity reached 19.294 mS/cm after 7 days, a notable shift from the initial value of 18.746 mS/cm.

This rise in ionic conductivity likely results from the release of ions from the plant extract, as well as interactions between the silver ions and the SBF fluid. The release of H⁺ ions and other ionic components from the plant extract contributed to an increased solution ionicity, thereby enhancing its conductivity. Additionally, samples with higher extract contents displayed increased porosity and reduced density, facilitating a more rapid release of compounds from the hydrogel matrix into the SBF fluid. Greater porosity enhances fluid transport and compound leaching from within the hydrogel, accelerating the release of acidic substances and ions into the medium. The release kinetics of antioxidants, which correlated strongly with the extract concentration, further suggest that samples with more extract (e.g., 3_Ag, 4_Ag, 5_Ag) released greater quantities of bioactive compounds, especially phenolic substances. These compounds contributed to the acidification of the SBF fluid, with their gradual release causing concurrent decreases in the pH and increases in the ionic conductivity. Additionally, the presence of silver particles may have further influenced the ionic conductivity through ion exchange with the fluid.

### 2.7. Kinetics of Release of Substances with Antioxidant Activity

**Figure 12:**
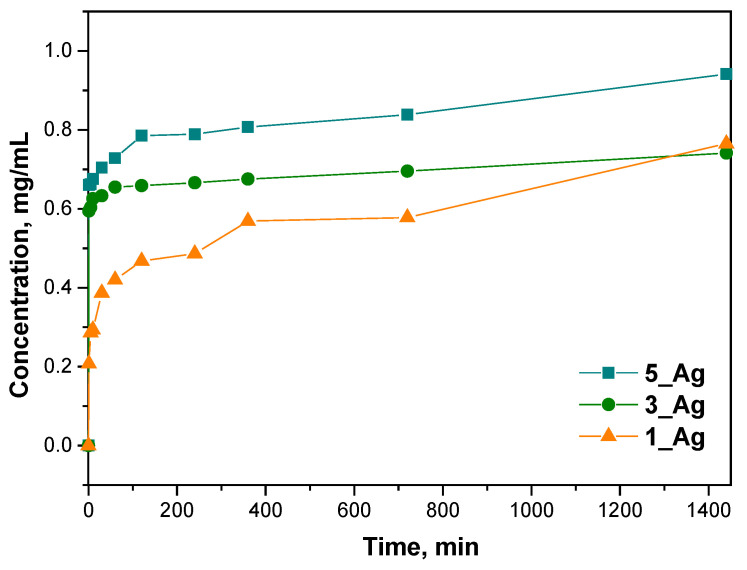
Correlation of the concentration of the released extract with time.

This study analyzed the release kinetics of compounds with antioxidant properties from hydrogels containing increasing amounts of plant extract with silver particles. Three samples were analyzed in the release study: 1_Ag, 3_Ag, and 5_Ag, with each successive sample containing a larger amount of suspended extract. The samples were selected to represent extreme (1_Ag and 5_Ag) and intermediate (3_Ag) concentrations of silver content, providing a comprehensive overview of the release behavior across the range of formulations. The concentration dependence of the released antioxidant substance as a function of time is presented in Figure 12.

At the outset of this study, the antioxidant properties of the plant extract used in synthesizing the silver particles were assessed. The antioxidant activity was measured at 197.78 ± 1.04 mg GAE/g, where GAE refers to the gallic acid equivalent. This indicates that the extract contains 197.78 mg of phenolic compounds (expressed as gallic acid) per gram of its dry weight, which contribute to its antioxidant potential. Gallic acid served as the calibration standard in our application of the Folin–Ciocalteu method, enabling comparison of the antioxidant capacities across samples based on their phenolic contents. These findings confirm that the extract utilized in the synthesis exhibits substantial antioxidant activity, a property that is particularly advantageous for biomedical applications, where antioxidants are essential for protecting materials against oxidative stress. It is worth noting that the arnica flower used was a commercial product with chemical characteristics that have been detailed in other works, e.g., Rywaniak et al., and the results obtained in the presented work correspond to previous findings [52]. Similarly, recent research on *Mansoa alliacea* extracts demonstrates the potential of plant-derived silver nanoparticles to retain and amplify the therapeutic properties of their source material, suggesting that such eco-friendly synthesis approaches can enhance the biomedical utility of silver particles while maintaining their biocompatibility and reducing toxicity [53].

**Figure 13:**
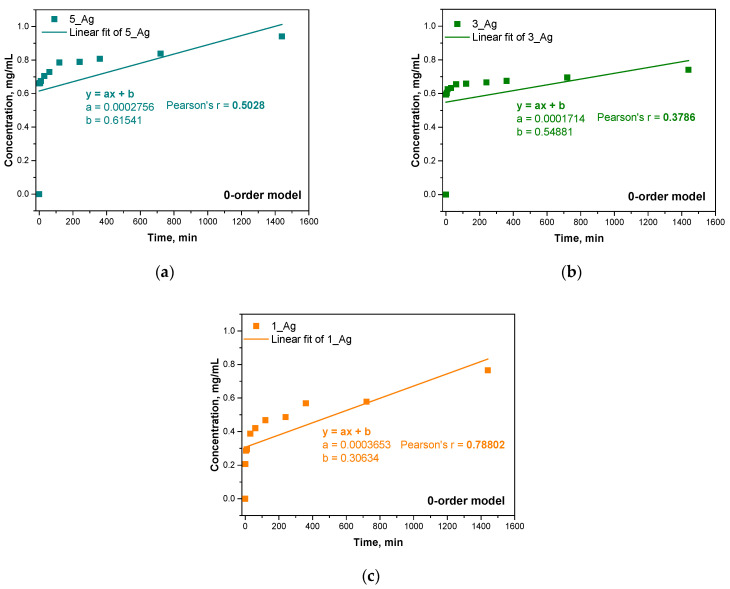
Linear fit of experimental results for zero-order kinetic model for samples 5_Ag (**a**), 3_Ag (**b**), 1_Ag (**c**).

**Figure 14:**
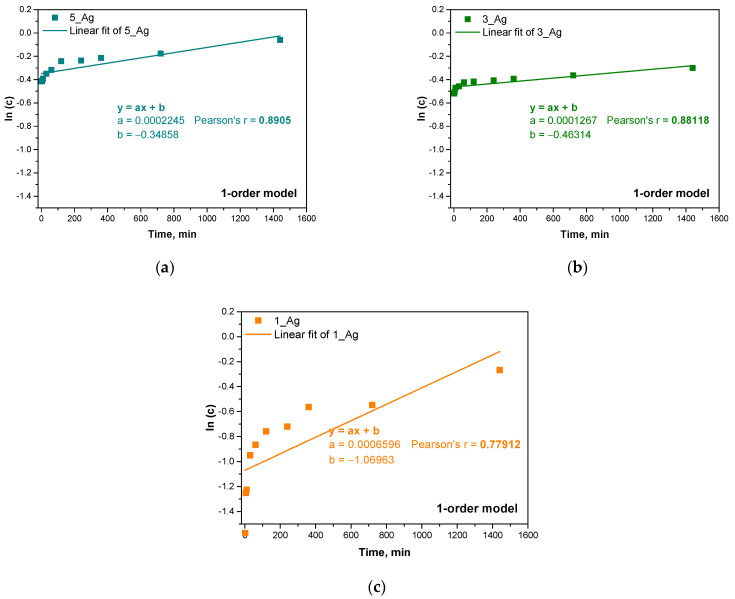
Linear fit of experimental results for first-order kinetic model for samples 5_Ag (**a**), 3_Ag (**b**), 1_Ag (**c**).

**Figure 15:**
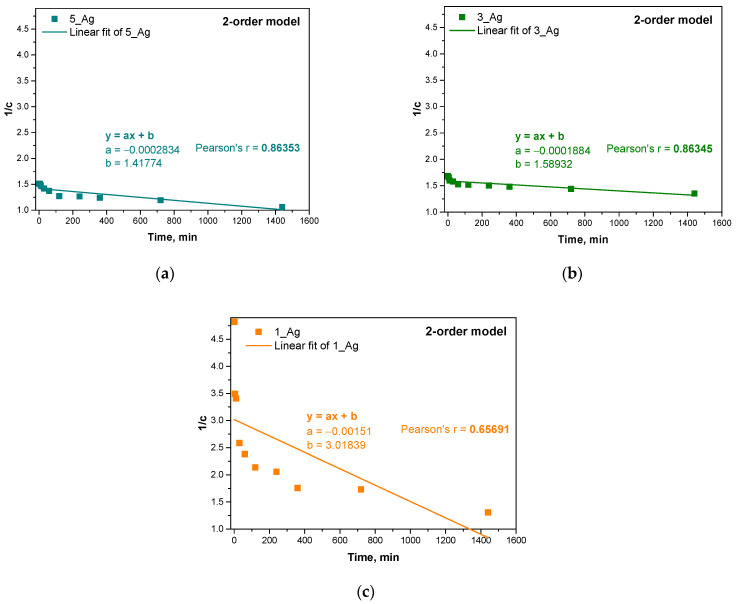
Linear fit of experimental results for second-order kinetic model for samples 5_Ag (**a**), 3_Ag (**b**), 1_Ag (**c**).

Next, three kinetic models were used: a zero-order model, a first-order model, and a second-order model. The results of fitting these models are presented in the form of graphs, and Pearson’s correlation coefficients (r) were calculated for each model with respect to the experimental data. The results are presented below in Figure 13, Figure 14 and Figure 15 and Table 6.

The samples with higher contents of plant extract (5_Ag and 3_Ag) showed more complex mechanisms for the release of antioxidant compounds, which was best described by first- and second-order kinetic models. This means that the release process in these samples is concentration-dependent and may be subject to interactions between the hydrogel components and silver particles. A higher amount of extract results in a more dynamic release, which is related to the reactive nature of the bioactive compounds contained in the extract.

Sample 5_Ag demonstrated the highest alignment with a first-order kinetic model (r = 0.8905), indicating that, in this sample, the release of substances is primarily influenced by the extract concentration within the hydrogel. This may be due to the larger amount of suspension containing silver particles and antioxidant compounds. In contrast, for sample 1_Ag, which has the lowest suspended solids content, the zero-order model provided the best fit (r = 0.78802), suggesting a more linear release pattern with less dependency on the bioactive substance concentration. Here, the release occurs at a steady rate, likely indicating a more predictable depletion of active compounds. This suggests that a lower extract concentration promotes a simpler, more consistent release profile. The analysis reveals that the studied release mechanisms are highly influenced by the concentrations of the silver particle suspension and plant extract. In samples with higher concentrations (e.g., 5_Ag and 3_Ag), the release process is more dynamic and concentration-dependent, whereas, in lower-concentration samples (e.g., 1_Ag), the release is more uniform and linear, suggesting reduced interactions within the system components. The increased complexity of the release mechanisms of samples with higher concentrations of silver particles and extract may be advantageous for applications requiring a controlled, gradual release of active ingredients. Conversely, hydrogels with lower extract concentrations, like sample 1_Ag, may be better suited for applications that require a more consistent and predictable release. These release kinetics findings align with prior analyses of hydrogel properties such as porosity and water vapor transmission rate (WVTR), where increased porosity, associated with higher extract and silver particle concentrations, contributes to more complex release mechanisms for antioxidant compounds, which is reflected in the stronger fits to first- and second-order models. 

The different release mechanisms in hydrogel systems, which depend on the amount of extract and silver particles, indicate the complexity of the kinetic process in the system. These results provide important information that can be used to optimize hydrogels in the context of the controlled release of bioactive substances in various applications, including biomedical applications such as in wound dressings and drug delivery systems.

According to studies by other researchers, the first-order kinetic model can be effectively used to describe the process of drug release from polymeric membranes, suggesting that the mechanism depends on the concentration of the substance. Accordingly, the drug release was proportional to the amount of remaining substance in the matrix. The amount of drug released decreased as the concentration gradient decreased over time. This process was mainly due to the diffusion of the medium into the composite matrix [54]. It should be taken into account that the kinetics of drug release can proceed in different stages according to different models. Usually, these are very complex relationships that require checking for more mathematical fits. 

Drug release models are often complex, requiring the consideration of various mathematical relationships to provide an accurate description. This may involve examining diverse mechanisms and models, such as those based on diffusion, erosion, or matrix swelling. However, the results that are obtained still allow for significant conclusions regarding the release mechanism. The simplified models employed in this analysis offer valuable insights into the differences in release mechanisms that depend on the quantity of the modifying additive. The findings of this study indicate that the release mechanism shifts as the modifier content increases, aligning with the observed physicochemical properties of the samples, such as their porosity, density, and water vapor permeability. This consistent correlation between the release kinetics and the structural attributes of the material highlights the modifier’s impact on the system’s overall properties.

## 3. Materials and Methods

### 3.1. Materials

Polyvinylpyrrolidone (PVP, powder, average mol wt. 10,000), diacrylate poly(ethylene glycol) (crosslinking agent, PEGDA, average molecular weight Mn = 700 g/mol), 2-hydroxy-2-methylpropiophenone (photoinitiator, 97%, d = 1.077 g/mL), silver nitrate (powder, anhydrous), acetone (ACS reagent, ≥99.5%), chloroform (≥99.9% (GC)), and DMSO (ACS reagent, ≥99.9%) were purchased from Sigma Aldrich (Saint Louis, MO, USA). In turn, dried *Arnica montana* flowers were purchased from Kawon (Gostyn, Poland).

### 3.2. Synthesis of Silver Particles via Extracts from A. montana Flowers

Silver particles were synthesized by reducing silver nitrate (AgNO_3_) with arnica flower extract, which acted as a natural reducing agent. Commercially available dried *Arnica montana* flowers, supplied by Kawon–Hurt (Gostyń, Poland), were used to prepare the extract, following the method described by Rywaniak et al. In this approach, the dried flowers were extracted using an acetone–water solution, followed by centrifugation and filtration to remove solid residues. The resulting extract was concentrated under reduced pressure to obtain a polyphenol-rich solution suitable for use in further synthesis. A detailed description is provided in [52]. Next, a 5 mM silver nitrate solution was prepared and combined with the pre-prepared flower extract at a 1:9 volume ratio. The reaction took place at room temperature under continuous stirring with a magnetic stirrer. Active compounds in the extract facilitated the reduction of silver ions (Ag⁺) to elemental silver (Ag^0^), resulting in a visible color change in the solution. The reaction proceeded for 45 min, after which the silver particles were isolated by centrifugation and rinsed with distilled water to eliminate any residual reactants. Finally, the particles were resuspended in a fresh portion of the plant extract.

### 3.3. Characteristics of Silver Particles

The synthesized particles were analyzed for size distribution using the Anton Paar PSA 1190 analyzer. To enhance result accuracy, measurements were repeated five times. This analysis was conducted on the particle suspension within the plant extract at room temperature. Additionally, to determine the elemental composition of the resulting suspension, X-ray fluorescence analysis was performed. This was achieved using a Bruker S2 Puma Energy Dispersive X-ray fluorescence spectrometer.

### 3.4. Synthesis of Polymeric Systems Containing Silver Particles

Polymeric materials modified with silver particles suspended in a plant extract were obtained by photopolymerization. For this purpose, a 15% solution of polyvinylpyrrolidone, which was the basic component of these materials, was used. The PVP solution was mixed with an appropriate amount of suspended particles, and then a crosslinking agent (PEGDA with a molecular weight of 700 g/mol) and a photoinitiator (2-hydroxy-2-methylpropiophenone) were added. The mixture was subjected to homogenization using a mechanical homogenizer to ensure uniform dispersion of the silver particles throughout the composition. The compositions of the obtained polymer compositions are presented in Table 7.

**Figure 16:**
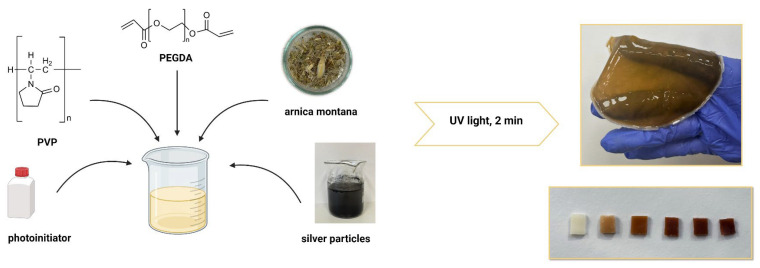
Scheme of synthesis of polymer systems.

Photopolymerization was carried out using an EMITA-60 lamp with a power of 180 W and a wavelength of 320 nm. Time of photopolymerization was 120 s. After photopolymerization, the samples were dried in a forced-air oven for 24 h at a temperature of 60 °C. The scheme for synthesizing polymer systems enriched with silver particles is presented in Figure 16.

### 3.5. FT-IR Infrared Spectroscopy Analysis

The purpose of FT-IR spectroscopic analysis in this study was to identify specific functional groups in hydrogels that were been modified with silver particles suspended in *Arnica montana* flower extract. This study provides an understanding of how the addition of silver and active ingredients from arnica affect the chemical and structural properties of hydrogels. The analysis was performed using a Nicolet iS5 spectrometer from Thermo Scientific (Loughborough, UK). Spectra were recorded in the range of 4000–500 cm^−1^, with a resolution of 4.0 cm^−1^, under room temperature conditions.

### 3.6. Analysis of Physicochemical Properties

The physicochemical analysis of hydrogels focused on evaluating their sorptive capacities. The aim was to investigate the potential of hydrogel dressings to effectively absorb therapeutic fluids. The process began with the preparation and drying of hydrogel samples, which were then accurately weighed on a Radwag analytical balance. The samples were then placed in three different incubation fluids: simulated body fluid (SBF), Ringer’s solution, and distilled water. The samples were incubated for different periods of time: 1 h, 12 h, and 24 h. After each incubation step, the excess liquid was removed and the weight of the samples was measured again. Based on this, the swelling coefficient was calculated using the equation:

(1) $$\alpha =\frac{\left(m_{t}-m_{0}\right)}{m_{0}}$$

where:

*α—*degree of swelling of the sample, g/g;
*m_t_—*mass of swollen hydrogel after time *t*, g;
*m*_0_*—*initial hydrogel mass, g.

Each sample was subjected to three repetitions, and the obtained swelling coefficient values were averaged to obtain representative data on the sorption capacity of hydrogel matrices.

The incubation solutions, Ringer’s solution, and simulated body fluid (SBF) were prepared according to the compositions specified in the tables below. The solutions were prepared by adding the components in the specified order, ensuring that each component was completely dissolved before adding the next one. The SBF solution was prepared at a constant temperature of 36 ± 0.5 °C to maintain consistency and accuracy in composition. The chemical composition of the SBF fluid and Ringer’s solution is shown in Table 8 and Table 9.

In addition, to determine the porosity and density values of the hydrogels, the samples were immersed in a certain volume of isopropanol. After 5 min, the change in the volume of alcohol absorbed by the sample was measured. The sample was then removed from the isopropanol, and the difference in the volume of alcohol before and after removing the sample was used to calculate the density and porosity according to the following equations:

(2) $$d=\frac{m}{V_{2}-V_{3}}$$

(3) $$P=\frac{V_{1}-V_{3}}{V_{2}-V_{3}}$$

where:

*m—*mass of the investigated sample, g;
*V*_1_*—*initial volume of isopropanol, cm^3^;
*V*_2_*—*volume of isopropanol with immersed sample, cm^3^;
*V*_3_*—*volume of isopropanol after sample removal, cm^3^.

### 3.7. Water Vapor Transmission Rate

Assessing the water vapor transmission rate (WVTR) of hydrogels is essential for understanding their water vapor permeability, a key property for medical applications. Hydrogel samples, shaped into disks with specified weights and thickness, were positioned as covers over glass containers holding a known weight of water. These setups were then incubated at 37 °C for 24 h. After this incubation period, the water in each container was re-weighed. WVTR was determined using the following formula:

(4) $$WVTR=\frac{m_{0}-m_{t}}{A\;\times \;t}$$

where:

*WVTR—*water vapor transmission rate, g/m^2^h;
*m*_0_*—*initial mass of water, g;
*m_t_—*mass of water after time *t*, g;
*t—*time, h;
*A—*area of evaporation by the sample, m^2^.

### 3.8. Evaluation of Surface Roughness and Microscopic Observations

To investigate the surface and structure of the synthesized polymer matrices, observations were conducted using an advanced VKX-700 Keyence digital microscope (KEYENCE INTERNATIONAL, Mechelen, Belgium). The primary objective of this analysis was to gain a detailed understanding of the surface morphologies of hydrogels modified with silver particles suspended in *Arnica montana* flower extract. This examination enabled the assessment of roughness profiles, crucial for evaluating the adhesive properties of these materials and their interactions with their surrounding environment. In addition, microscopic observations of the surface were carried out and then the obtained images were analyzed. 

### 3.9. Incubation in Simulated Body Fluids

The incubation analysis aimed to assess the interactions between the hydrogel matrix and solutions simulating human physiological fluids. Variations in pH values may indicate the leaching of uncrosslinked components, potentially impacting sample stability within the analyzed solutions. Hydrogel samples, each 1 cm in diameter, were placed in sterile containers containing 50 mL of SBF solution. Incubation was maintained at a controlled temperature of 37 °C, with pH values monitored over 7 days using a CX-701 ELMETRON multifunctional instrument (ELMETRON, Zabrze, Poland). Additionally, ionic conductivity measurements were conducted in parallel using the same multifunctional instrument (CX-701 ELMETRON), which was equipped with both a pH electrode and a conductivity electrode, enabling simultaneous measurements.

### 3.10. Kinetics of Release of Substances with Antioxidant Activity

To assess the release kinetics of antioxidant compounds, the Folin–Ciocalteu method was employed, enabling quantification of phenolic compounds in the extracts. A calibration curve was first generated for gallic acid to establish a correlation between its concentration and absorbance. Subsequently, the release of phenolic compounds was measured at various time intervals: 1 min, 5 min, 30 min, 1 h, 12 h, and 24 h. The release experiments were conducted in simulated body fluid (SBF) at 37 °C. The samples varied in their concentrations of suspended particles, facilitating a comparative analysis between the samples with the highest and lowest additive contents. The total phenolic content of the plant extract was expressed as gallic acid equivalent (mg GAE/g) to quantify antioxidant capacity. The concentration of the released phenolic compounds (mg/mL) was determined spectroscopically, and release kinetics were analyzed using three standard kinetic models according to the following equations:

(5) $$c=c_{0}-k_{0}t$$

(6) $$\ln\left(c\right)=\ln\left(c_{0}\right)-k_{1}t$$

(7) $$\frac{1}{c}=\frac{1}{c_{0}}+k_{2}t$$

where:

*c*—concentration of the substance;
*k*_0—_kinetic constant in the 0-order model;
*k*_1—_kinetic constant in the 1-order model;
*k*_2—_kinetic constant in the 2-order model;
*t*—time.

The kinetic models of zero-order, first-order, and second-order are widely applied in the study of active substance release to elucidate the underlying mechanisms of delivery systems. Zero-order kinetics, characterized by a constant release rate, is commonly associated with systems designed for sustained and controlled release, ensuring uniform therapeutic effects over time [55,56]. In contrast, first-order kinetics, where the release rate depends on the concentration of the active substance, is frequently observed in diffusion-controlled systems [57]. Second-order kinetics, less commonly reported, often reflects more complex interactions between the active substance and the matrix [58]. These models are foundational in release studies, aiding in the characterization and optimization of delivery systems, including hydrogels and polymer-based matrices. Their consistent use across studies underscores their reliability in describing and predicting release behaviors for bioactive compounds [59].

## 4. Conclusions

- Increasing the silver particle concentration in hydrogels resulted in a heightened surface roughness, as indicated by the parameters Ra and Rz. For the control sample without silver particles (0_Ag), the Ra was 8.42 µm, while for the sample with the highest silver content (5_Ag), the Ra increased significantly to 16.33 µm. Similarly, the Rz value rose from 46.47 µm in 0_Ag to 59.78 µm in 5_Ag. This enhanced roughness may positively influence the mechanical and biophysical properties of hydrogels, particularly in terms of cell and fluid interactions, which are essential for biomedical applications.
- The incorporation of silver particles elevated the water vapor transmission rate (WVTR), with a pronounced effect being observed at higher silver concentrations. For the control sample (0_Ag), the WVTR was 65.169 g/m^2^h, while for the sample with the highest silver content (5_Ag), the WVTR increased to 93.772 g/m^2^h. The elevated WVTR suggests that hydrogels with increased silver content have enhanced moisture regulation capabilities, which is advantageous in wound dressing applications.
- A decrease in the hydrogel density accompanied by an increase in porosity was observed with rising silver particle levels. The density of the hydrogel decreased from 0.6669 g/cm^3^ in sample 0_Ag to 0.2963 g/cm^3^ in sample 5_Ag. Concurrently, the porosity rose from 4% to 11.04% across the same range. This increased porosity is associated with improved water vapor permeability and greater surface roughness, indicating a close relationship between the hydrogel’s internal structure and its properties. These findings align with our sorption capacity analysis, showing that higher porosity and lower density correlate with an increased sorption potential.
- The reductions in density, alongside increases in the porosity, roughness, and water vapor permeability, suggest that the addition of silver significantly modifies hydrogels’ structures and functional attributes. Enhanced porosity and roughness improve the material’s moisture management and its interaction with the biological microenvironment, key factors for biomedical use.
- Release kinetics analysis revealed that the release mechanism of bioactive substances is influenced by the concentration of the modifying agent. The simplified models that we applied differentiated these release processes, and the changes in release kinetics were consistent with the observed physicochemical properties, reinforcing the effect of the modifier on the hydrogel’s structure and functionality.
- The integration of silver particles into hydrogels not only modifies their structural and functional attributes but also provides a versatile platform for biomedical applications, particularly in wound care. The ability to tailor the porosity, surface roughness, and moisture regulation of these materials enables precise control over the release kinetics of bioactive compounds, enhancing their therapeutic efficacy. This study underscores the unique synergy between the hydrogel matrix and silver particles, offering a sustainable and customizable material with potential for further functionalization, such as the incorporation of additional bioactive agents. Future work will focus on optimizing these systems for targeted applications, exploring their long-term stability and evaluating their in vivo performance to expand their utility in advanced medical treatments.

## Figures and Tables

**Table 1:** Results of particle size analysis of obtained Ag particles.

	D_10_, µm	D_50_, µm	D_90_, µm	Mean Size, µm
Mean value	0.04651	0.2351	2.211	0.7816
Standard deviation	0.00741	0.00310	0.07869	0.02798

**Table 2:** Element composition of Ag suspension.

Element	Concentration, %
Ag	96.03
Ca	1.57
Cl	1.34
Si	0.39
Al	0.27
Mg	0.23
S	0.11
P	0.06

**Table 3:** Summary of absorption bands of hydrogel materials.

Range of Wave Number, cm^−1^	Functional Group/Chemical Compound	Component
3400–3500	O–H stretching (hydroxyl groups)	Water, PVP, phenolic compounds from plant extract
2950–3000	C–H stretching (alkyl groups)	PVP, PEGDA, organic compounds from extract
1650–1700	C=O stretching (carbonyl bonds)	PVP, PEGDA, photoinitiator, flavonoids from extract
1450–1500	C–H bending (methyl and methylene groups)	PVP
1250–1300	C–N stretching	PVP
1100–1150	C–O–C stretching (ester bonds)	PEGDA, organic compounds (e.g., carboxylic acids)
1000–1100	C–O stretching (alcohol and phenolic groups)	Phenolic compounds from plant extract
700–900	C–H out-of-plane bending (aromatic bonds)	Photoinitiator, aromatic compounds from extract

**Table 4:** Statistical analysis of obtained data based on the two-way analysis of variance (ANOVA) (for sorption ability after 1 h).

Independent Variable	Sum of Squares	Mean Square	f-Value	*p*-Value
Type of incubation fluid	2.73379	1.3669	597.47339	2.45 × 10^−28^
Composition of sample	0.65438	0.13088	57.20591	4.4408 × 10^−16^
Interaction	0.06489	0.00649	2.8364	0.01049

At the 0.05 level, the population means of “type of incubation fluid” are significantly different. At the 0.05 level, the population means of “composition of sample” are significantly different. At the 0.05 level, the interaction between both factors is significant.

**Table 5:** Summary of roughness parameters.

Sample Name	Ra, µm	Rz, µm
0_Ag	8.42	46.47
1_Ag	10.61	67.33
2_Ag	13.95	50.19
3_Ag	13.68	54.01
4_Ag	12.79	63.92
5_Ag	16.33	59.78

**Table 6:** Pearson’s r correlation coefficient for different kinetic models.

Sample Name	Pearson’s r Correlation Coefficient		
	0-Order Model	1-Order Model	2-Order Model
5_Ag	0.5028	0.8905	0.86353
3_Ag	0.3786	0.8118	0.86345
1_Ag	0.78802	0.77912	0.65691

**Table 7:** Compositions of polymeric systems.

PVP Solution, mL	Fotoinitiator, mL	Crosslinking Agent, mL	Ag-Particles Suspension, mL	Sample Code
20	0.15	3.0	0	0_Ag
			1.0	1_Ag
			2.0	2_Ag
			3.0	3_Ag
			4.0	4_Ag
			5.0	5_Ag

**Table 8:** Composition of Ringer’s solution.

	Component	Amount [g/L]
1	NaCl	8.600
2	KCl	0.300
3	CaCl_2_·H_2_O	0.480

**Table 9:** Composition of SBF.

	Component	Amount [g/L]
1	NaCl	8.035
2	NaHCO_3_	0.355
3	KCl	0.225
4	K_2_HPO_4_·3H_2_O	0.231
5	MgCl_2_·6H_2_O	0.311
6	1M HCl	39 mL
7	CaCl_2_	0.292
8	Na_2_SO_4_	0.072
9	Tris	6.118

## Data Availability

The data that support the findings of this study are contained within the article.
